# Inhibitory synaptic plasticity: spike timing-dependence and putative network function

**DOI:** 10.3389/fncir.2013.00119

**Published:** 2013-07-18

**Authors:** T. P. Vogels, R. C. Froemke, N. Doyon, M. Gilson, J. S. Haas, R. Liu, A. Maffei, P. Miller, C. J. Wierenga, M. A. Woodin, F. Zenke, H. Sprekeler

**Affiliations:** ^1^Department of Physiology, Anatomy, and Genetics, Centre for Neural Circuits and Behaviour, University of OxfordOxford, UK; ^2^School of Computer and Communication Sciences and School of Life Sciences, Brain Mind Institute, École Polytechnique Fédérale de Lausanne (EPFL)Lausanne, Switzerland; ^3^New York University School of Medicine Skirball Institute of Biomolecular MedicineNew York, NY, USA; ^4^Division of Cellular and Molecular Neuroscience, Centre de Recherche Université Laval Robert-GiffardQuébec, QC, Canada; ^5^Lab for Neural Circuit Theory, Riken Brain Science InsituteWako-shi, Saitama, Japan; ^6^Department of Biological Sciences, Leigh UniversityBethlehem, PA, USA; ^7^Department of Biology, Emory UniversityAtlanta, GA, USA; ^8^Department of Neurobiology and Behavior, Stony Brook UniversityStony Brook, NY, USA; ^9^Volen Center for Complex Systems, Brandeis UniversityWaltham, MA, USA; ^10^Division of Cell Biology, Faculty of Science, Utrecht UniversityUtrecht, Netherlands; ^11^Department of Cell and Systems Biology, University of TorontoToronto, ON, Canada; ^12^Institute for Theoretical Biology, Humboldt-Universität zu BerlinBerlin, Germany

**Keywords:** GABAergic transmission, spike timing-dependent plasticity, electrophysiology, modeling and simulations, network dynamics and function, chloride dynamics

## Abstract

While the plasticity of excitatory synaptic connections in the brain has been widely studied, the plasticity of inhibitory connections is much less understood. Here, we present recent experimental and theoretical findings concerning the rules of spike timing-dependent inhibitory plasticity and their putative network function. This is a summary of a workshop at the COSYNE conference 2012.

In the decades since Donald Hebb suggested that associative learning could rely on changes in the strength of neuronal connections (Hebb, 1949; Martin et al., 2000), synaptic plasticity has been a major research field in neuroscience. Studies of plasticity have primarily focused on synaptic connections between excitatory pyramidal cells, because excitatory-to-excitatory (EE) connections are most prevalent in cortex and form a relatively homogeneous population. The plasticity of any other type of connection has, until recently, attracted significantly less attention, mainly because of experimental obstacles in their study. With the advent of fluorescent labeling and optical manipulation of neurons according to their genetic type (Deisseroth et al., 2006; Miesenböck, 2011; Cardin, 2012), the nature and plasticity of these connections has moved into the focus of current research. Here we summarize recent advances in the emerging field of inhibitory-to-excitatory (IE) plasticity that were presented at a workshop at the COSYNE conference in early 2012.

Inhibitory cells make up roughly 20% of all cortical neurons and consist of many different cell types (Markram et al., 2004; Klausberger and Somogyi, 2008). Their function is thought to be equally heterogeneous, ranging from providing global stability to neuronal dynamics to temporal selection mechanisms that control spike timing in single neurons and the degree of neuronal synchronization (Moore et al., 2010; Isaacson and Scanziani, 2011). Additional computational functions comprise input separation through winner-take-all wiring schemes or context-dependent widening of the dynamic range of neuronal responses (Carvalho and Buonomano, 2009). Given this rich set of potential computational functions of inhibition, the plasticity of inhibitory connections is of great interest, because it controls the efficacy of any of these mechanisms. We were interested specifically in the spike-dependent rules that govern inhibitory plasticity (both in weight and structure) and in their functional effects. Given the different functional roles inhibitory neurons could play in sensory processing and network dynamics, it is not surprising that the results presented here are often conflicting. More comprehensive reviews on other aspects of inhibitory synaptic plasticity (e.g., the underlying molecular machinery) can be found elsewhere (Lamsa et al., 2010; Woodin and Maffei, 2010; Castillo et al., 2011; Kullmann et al., 2012).

## Structural plasticity of inhibitory connections

Throughout life and development, synaptic inputs are formed at distances of tens to hundreds of micrometers from the soma (Terauchi and Umemori, 2012). Inputs stemming from these synapses are often integrated with one another before they reach the soma and ultimately evoke (or fail to evoke) action potentials. It was recently suggested that dendrites act as independent computational units (Poirazi et al., 2003; Losonczy et al., 2008; Branco and Häusser, 2010) that locally regulate many important cellular processes, such as plasticity and protein synthesis. In this light, a dendritic, local regulation of excitatory and inhibitory synapses should exist and be actively maintained during synaptic plasticity (Liu, 2004; Liu et al., 2007; Bourne and Harris, 2011).

The formation of excitatory synapses is often mediated by the outgrowth of small dendritic protrusions and synapses can thus form between two neurons that previously had no physical interaction. Using two-photon microscopy, Wierenga and colleagues recently showed that inhibitory synapses are formed in a fundamentally different way (Wierenga et al., 2008). New inhibitory synapses grew in locations where an inhibitory axon is already in close contact with a postsynaptic dendrite. The question emerges of what determines the timing and location for the creation of a new inhibitory synapse along the inhibitory axon.

One possibility is that coordinated forms of pre- and postsynaptic activity could play a role in these processes, perhaps even at the level of individual boutons and spines that intersect or are in close proximity to each other. Wierenga studied inhibitory plasticity by monitoring the dynamic behavior of the inhibitory boutons along the axon in hippocampal organotypic cultures of transgenic mice using two-photon microscopy, and showed that even during baseline activity inhibitory boutons are highly dynamic (Schuemann et al., 2013). Roughly 80% of inhibitory boutons were present during the entire 4–5 h imaging period, most likely reflecting stable inhibitory synapses. The other boutons showed highly dynamic behavior. Boutons appeared, disappeared and reappeared at specific locations along the inhibitory axons, presumably axon-dendrite crossings. In addition, these dynamic boutons showed large variance in their size. This indicates active trafficking of presynaptic material, and competition between neighboring boutons along inhibitory axons, similar to what was previously shown for excitatory axons (Staras, 2007). It also suggests that inhibitory axons are continuously exploring potential locations for the formation of new synaptic contacts.

**Figure 1:**
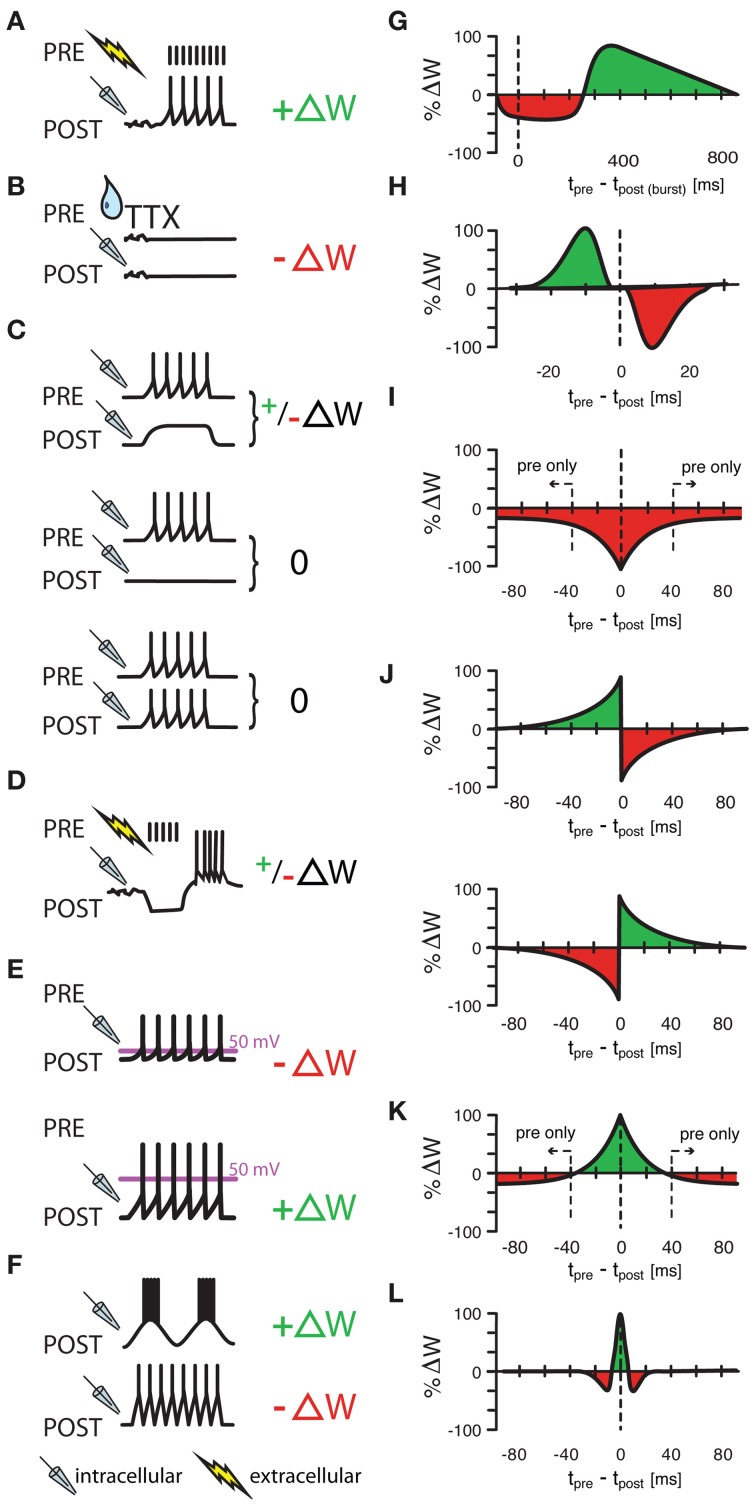
**Various protocols of ISP induction. (A)** Homeostatic plasticity induced by Hartmann et al., (2008) in >4 week old mouse CA1 hippocampal slices. A one second long extracellular stimulus of 100 Hz, delivered in the presence of glutamatergic and GABA_B_ receptor blockers provoked a strengthening of GABAergic synapses through increased presynaptic GABA concentrations. **(B)** A similarly homeostatic response was induced by Kilman et al., (2002) in cultures of P3–P5 rat visual cells. Here, 2 day long silencing of the culture with TTX led to decreased amplitude of inhibitory post synaptic potentials (and loss of synapses) that was mediated by a decrease in GABA_A_ receptors. **(C)** Congruent with **(A,B)**, Maffei et al., (2006) showed that a postsynaptic depolarization in the presence of presynaptic bursts (20 bursts of ten presynaptic action potentials at 50 Hz) strengthens synapses in slices of P21 rat visual cortex with normal activity, but weakens synapses in previously monocularly deprived animals [and thus slices with consequently lower baseline activity and presumably already potentiated inhibitory synapses, **(C)**, upper panel]. Presynaptic bursts, coupled with postsynaptic silence or firing did not induce any change at all [**(C)**, middle and lower panel, respectively]. **(D–F)** Other protocols had “non-homeostatic” effects: Aizenman et al., (1998) induced synaptic weight changes (with 10 spike bursts at 2 Hz) in inhibitory synapses in P11–P15 coronal slices of rat cerebellum that were dependent on the postsynaptic firing frequency of the inhibitory rebound burst **(D)** and Kurotani et al., (2008) could control ISP in slices of layer V primary visual cortex of P20–P30 rats by altering either the postsynaptic rest potential during intracellular (sole postsynaptic) stimulation (15 5s-bursts at 20 Hz) **(E)**, or by modifying the frequency of postsynaptic sub-threshold membrane fluctuations **(F)**. Additionally to these non-spike timing-dependent protocols, three experimental studies **(G–I)** have shown spike-timing dependence under certain conditions. Holmgren and Zilberter, (2001) successfully manipulated the amplitude of synaptic weight changes in P14—P16 somatosensory cortex slices of rat by pairing postsynaptic bursts (25–40 times 10 spikes at 50 Hz) with single presynaptic spikes at up to 800 ms after the onset of the burst **(G)**. Conversely, Haas et al., (2006) found bidirectional plasticity windows **(H)** on timescales more reminiscent of the classical excitatory STDP window in P14—P21 rat slices of entorhinal cortex and Woodin et al., (2003) found monodirectional plasticity in rat hippocampus cultures and slices. Interestingly, temporally proximal spike pairs weakened synaptic efficacy (measured from rest) through local changes in chloride reversal but sole presynaptic events decreased the amplitude of synaptic conductance **(I)**. **(J–L)** Other learning rules have been tested in models, but have not been observed in experiments. Luz and Shamir, (2012) used Hebbian and Anti-Hebbian variations of classical, asymmetric STDP windows **(J)**, as well as a symmetric form of iSTDP **(K)** also used by Vogels et al., (2011) that lead to strengthened synapses for near coincident spike pairs, but to weakened synapses for sole presynaptic events. Gilson et al., (2012) used a similar, mexican-hat shaped learning rule to produce experimentally observable frequency response behaviors **(L)**. ΔW stands for a change in synaptic weight. In panel **(A–F)** a drop symbolizes the use of TTX; a flash symbolizes the use of an extracellular, and a pipette the use of an intracellular electrode. All values in **(G–L)** have been normalized to the maximum value of each data set.

Once a connection between neurons is established, the strength of the synapse remains to be tuned according to its function. In EE connections such tuning often happens through activity-dependent changes, the sign and degree of which crucially depends on the timing of pre- and postsynaptic spiking (Markram et al., 1997; Bi and Poo, 1998; Dan and Poo, 2004). Several studies address the question whether similar rules apply to inhibitory connections (Figure 1). We have separated their results here by the proposed function of the effect.

## Inhibitory plasticity can alter stimulus selectivity

In a previous study of inhibitory plasticity (although not necessarily iSTDP), Tao and Poo (2005) examined the organization of excitatory and inhibitory spatial receptive fields during development of the Xenopus optic tectum. Initially in young animals (e.g., stage 44 tadpoles), the spatial extent of synaptic receptive fields were quite broad, both for excitation and inhibition; however, excitatory and inhibitory fields were somewhat mismatched and had substantial non-overlapping regions. Over development, synaptic receptive field size was reduced, and excitatory and inhibitory fields became similar, in a manner that seemed to require specific temporal patterns of activity of tectal neurons.

Another example of inhibitory plasticity governing receptive field organization is the self-balancing of excitation and inhibition along sensory processing pathways in auditory cortex. Excitatory-inhibitory balance is a fundamental property of cortical networks, important for control of spike generation, information processing, synaptic plasticity, and prevention of epilepsy (Van Vreeswijk and Sompolinsky, 1996; Moore and Nelson, 1998; Wehr and Zador, 2003; Vogels and Abbott, 2005; Higley and Contreras, 2006; Froemke et al., 2007; de la Rocha et al., 2008; Monier et al., 2008; Okun and Lampl, 2008; Vogels and Abbott, 2009; Cafaro and Rieke, 2010). Recent results in a number of sensory systems indicate that in mature cortex, the strengths and response profiles of inhibitory inputs are in proportion to the strengths and profiles of excitatory inputs (Wehr and Zador, 2003; Higley and Contreras, 2006; Cafaro and Rieke, 2010; House et al., 2011; Saar et al., 2011). Specifically, in adult rat primary auditory cortex (A1), synaptic frequency tuning curves for excitation and inhibition are generally co-tuned and highly correlated [average correlation coefficient r: ≈0.7, Froemke et al. (2007)].

In contrast, in developing A1 of young rats Froemke et al. (2007) reported low correlation between excitation and inhibition. This was not due to a lack of inhibition. Inhibitory responses were present in young animals just after hearing onset, and the overall ratio of excitatory to inhibitory strengths seemed to be conserved in young and adult animals. Rather, the low correlation between excitation and inhibition reflected the untuned nature of inhibitory responses, similar to the observations in developing Xenopus tectum (Tao and Poo, 2005). While excitatory synaptic tuning curves were well-tuned and structured in young animals, inhibitory tuning curves were tuned randomly and generally broader in spectral extent. Changes in the pattern of sensory experience could accelerate the development of excitatory-inhibitory balance, and repetitive presentation of tones of a single frequency led to network-wide plasticity and adjustment of synaptic strength, calibrating excitation and inhibition and shifting the preferred frequency, due to an orchestrated set of long-term excitatory and inhibitory synaptic modifications (Dorrn et al., 2010).

Long-lasting shifts in preferred frequency could also be induced in adult A1, by pairing patterned sensory stimulation with direct activation of neuromodulatory centers. Froemke et al. (2007) focused on the effects of electrical stimulation of the cholinergic basal forebrain, involved in control of selective attention. Muscarinic receptor activation in A1 led to a transient disinhibition, breaking excitatory-inhibitory balance and gating induction of NMDA receptor-dependent synaptic plasticity. Excitatory tuning curves shifted toward the paired tone within ~10 min by two major mechanisms: enhancements of responses at the paired frequency, and decreases in responses at the original preferred frequency. Remarkably, over a longer timescale (several hours), inhibitory tuning curves shifted to re-balance the profiles of excitation, recovering excitatory-inhibitory balance (Froemke et al., 2007).

### Inhibitory plasticity after sensory deprivation

Maffei et al. (2006) showed that in primary visual cortex, inhibitory plasticity is induced to saturating levels quite rapidly after the onset of monocular deprivation (MD). This long-term potentiation of inhibition (iLTP) was produced when the presynaptic interneuron was active (i.e., spiking) while the postsynaptic excitatory cell was depolarized but inactive (Figure 1C). Further, postsynaptic spikes within a time window of approximately 20 ms of the pre-synaptic inhibitory spike would “veto” iLTP, preventing synaptic strengthening.

In a network with shared inhibition, such a plasticity mechanism could produce cross-inhibition between cells with different stimulus tuning, because weakly driven cells would allow strengthening of inhibitory synapses from cells activated by either the stimulus directly or stimulus-responsive excitatory cells, or both. In contrast, a cell that is strongly driven by a stimulus would veto the strengthening of its inhibitory inputs through postsynaptic spiking. Consequently, strongly active cells remain active, while weakly active cells reduce their activity, thus enhancing the contrast of the population response. Excitatory and inhibitory cells with similar stimulus tuning would not strengthen their inhibitory interaction, because they tend to be co-active. Conversely-tuned inhibitory cells on the other hand will strengthen their synapses and thus enhance contrast.

Bourjaily, Escobar and Miller tested these ideas in recurrent random and sparse networks of excitatory and inhibitory spiking neurons with two separate training paradigms. In the first paradigm, pairs of stimuli are presented successively, whereby input cells responding to each stimulus randomly connect to a fraction of cells in the recurrent network. They showed that so long as the veto by post-synaptic action potentials was incorporated, iLTP would strongly increase the selectivity to the overlapping stimulus-pairs, so as to enable accurate decision-making behavior in exclusive-OR (XOR) tasks (Bourjaily and Miller, 2011).

The second paradigm shows how direction selectivity to a moving visual stimulus can arise from the same plasticity rule. Inputs represent the activation of lateral geniculate nucleus (LGN) cells, each of which has a specific receptive field location and a specific lagâ i.e., a delay between visual stimulus activation and the time of neuronal spike emission. The random recurrent network here represents the initial state of cortical cells, each of which receives inputs from a random selection of LGN cells. The direction of the motion of a stimulus affects the temporal pattern of inputs but neither the number nor the strengths of input afferents. After 200 trials of random bidirectional training, cortical excitatory responses increase their direction selectivity as a consequence of iLTP. The resulting population distribution of direction selectivity resembles those measured in ferret after 16 h of training after eye-opening (Li et al., 2008).

### Inhibitory plasticity as a switch between excitatory LTP and LTD

The strong and rapid inhibitory plasticity reported in Maffei et al. (2006) suggests a close interaction with other experience-dependent events (Yoon et al., 2009) that have been reported in V1 after monocular deprivation.

A recent study by Wang et al. (2012) investigated this idea and indicates an entirely new function for inhibitory synaptic plasticity: determining the sign of activity-dependent plasticity at recurrent excitatory synapses. The same pattern of presynaptic activity induced depression or potentiation at recurrent excitatory synapses depending on the previous history of convergent inhibitory inputs. The tight relationship between excitatory and inhibitory plasticity, which was identified in acute slice preparation, occurs *in vivo* either as a consequence of manipulation of visual drive or following pharmacological potentiation of benzodiazepine sensitive inhibition (Maffei et al., 2010; Wang and Maffei, 2011; Wang et al., 2012). Recently, a similar switch-like function was also observed in corticostriatal pathways (Paille et al., 2013). In a functionally related study (Kurotani et al. 2008) showed that the sign of synaptic change in inhibitory synapses can be switched by contextually delivered Ca^2+^ signals (Kurotani et al., 2008).

Sensory perception is also strongly affected by changes in neuron and network states (Fontanini and Katz, 2008). The plasticity of synapses from fast spiking inhibitory neurons onto pyramidal neurons may reconfigure the state of excitatory neurons driven by the deprived eye and facilitate the functional changes that have been observed following sensory deprivation (Fagiolini et al., 1994; Frenkel and Bear, 2004). The results of Wang et al. (2012), Paille et al. (2013), and Kurotani et al. (2008) suggest that the interactions among neurons in a circuit are not merely the result of linear combination of changes that can be integrated in an additive or subtractive manner, but arise from the interaction of different neurons in the circuit and from the dynamics of their connectivity in response to sensory stimuli.

### Inhibitory plasticity can alter neuronal frequency selectivity

It has been suggested that different aspects of sensory information could be represented on different time scales of neural responses (Panzeri et al., 2010). For example, the rhythmic neuronal activity that has been observed in various areas of the brain (Buzsáki and Draguhn, 2004) may encode distinct information in different frequency channels. Decoding this information would then amount to the extraction of specific frequency components.

Single neurons with adjusted excitatory and inhibitory inputs can behave as such a band-pass filter (Bürck and van Hemmen, 2009). The filter properties crucially depend on both the time course and strength of the postsynaptic responses to excitation and inhibition. For typical synaptic time constants and delays, the neuronal response can exhibit a preferred frequency, or best modulation frequency (BMF), in the range between 10 and 200 Hz, in line with experimentally observed neuronal properties in the auditory midbrain (Krishna and Semple, 2000).

In a recent modeling study (Gilson et al., 2012) showed how inhibitory STDP can tune the BMF of a single neuron to its stimulating frequency. In their model, the neuron receives input spike trains from presynaptic neurons that share a common oscillatory firing rate modulation of a given “training frequency.” Excitatory synapses are fast, homogeneous and non-plastic. In contrast, inhibitory synapses are plastic according to a symmetric iSTDP rule (Figure 1L) and exhibit a broad range of time constants that are slower than the excitatory ones, arising e.g., from dendritic filtering. For a passive dendrite, the postsynaptic potentials (PSPs) arriving from a distal synapse at the soma are slower and delayed compared to that of a proximal synapse.

The inhibitory learning scheme is sensitive to the temporal correlations induced by the joint periodic rate modulation of the input firing rates. More precisely, Gilson and colleagues show that iSTDP potentiates different subsets of synapses depending for the presented training frequency, thus differentially changing the frequency response curve of the neuron. Under suitable conditions on the synaptic delays and PSP time constants the neuron learns its stimulating frequency in an unsupervised manner, i.e., the BMF matches the training frequency. This occurs when STDP potentiates proximal (distal) synapses for high (low) training frequency. This theory predicts that synapses responding to a given BMF form clusters on dendritic branches.

## Inhibitory synaptic plasticity can stabilize network dynamics

Haas et al. (2006) investigated spike timing-dependent plasticity of inhibitory synapses (iSTDP) in the entorhinal cortex, a brain area richly associated with spatial navigation (Hafting et al., 2005). Postsynaptic spikes were paired with extracellular stimulations that, in the presence of excitatory synaptic blockade, resulted in inhibitory postsynaptic potentials (IPSPSs). The amplitude of the inhibitory conductance was measured as the slope of the IPSP, before and after spike pairings. For presynaptic inputs preceding postsynaptic spikes, IPSPs were potentiated, with a maximal effect around Δ*t* = −10 ms (Δ*t* = *t*_pre_ −*t*_post_) of delay (Figure 1H). In other words, the postsynaptic spike had to arrive 10 ms after the presynaptic inhibitory spike for maximal strengthening. For inhibitory inputs that followed after the postsynaptic spike, a depression of synaptic strength was observed, with a peak effect near Δ*t* = + 10 ms of spike-input delay. Between these maxima, the observed change of synaptic efficacy was often bidirectional with no net change on average. Both potentiation and depression depended on calcium entry to the postsynaptic cell via L-type voltage-gated channels, presumably during the postsynaptic spike, similar to what has later been reported by Kurotani et al. (2008, cf. Figures 1E,F).

The functional implications of the observed iSTDP rule were explored in simulations of networks with dense and sparse connectivity (Haas et al., 2006). In densely connected feed-forward paths of excitatory neurons, so called synfire chains, a single interneuron was shown to successfully control runaway activity in the chain. Further, the rule scaled inhibitory strength according to the varying levels of excitatory strength and was self-stabilizing because once inhibition became strong enough, it prevented the postsynaptic spikes necessary to induce further strengthening. In a more elaborate network model with 400 excitatory and 100 inhibitory neurons and sparse, probabilistic connectivity, iSTDP in only a few synapses was sufficient to transform network-wide seizure-like activity into spatially restricted activity. When new inputs appeared at different locations, changes in synaptic strength tracked the input and grew to prevent seizure-like spread. These results show that even subtle changes in inhibitory strength can be sufficient to stabilize network dynamics.

For neocortical neurons, Vogels et al. (2011) and Luz and Shamir (2012) both hypothesized that iSTDP was the mechanism underlying the rebalancing of excitation and inhibition that occurs in A1 described above (Froemke et al., 2007). Vogels et al. (2011) studied a simplified plasticity rule, in which near-coincident pre- and postsynaptic spikes induced potentiation of the inhibitory synapse. Additionally, every presynaptic spike led to synaptic depression (Figure 1K). This learning rule was loosely based on various experimental results (Figures 1A–I) (Holmgren and Zilberter, 2001; Kilman et al., 2002; Woodin et al., 2003; Haas et al., 2006; Balena and Woodin, 2008; Hartmann et al., 2008; Woodin and Maffei, 2010), and used specifically to balance excitatory and inhibitory synaptic strengths. Vogels et al. (2011) simulated basic feed-forward networks with differentially tuned, static excitatory and untuned but plastic inhibitory synapses. Weak inhibitory synapses (those which failed to prevent *post*synaptic spiking in the immediate temporal proximity of a inhibitory *pre*synaptic spike and thus created spike pairs) were strengthened, and strong synapses (those which reliably prevented postsynaptic spikes, and thus produced only a pre-, but no postsynaptic spike) were weakened. Over time, this led to a precise, detailed balance of excitatory and inhibitory synaptic weights for each set of correlated excitatory and inhibitory signal channels as observed in Froemke et al. (2007) and others. In more general terms, the rule acts as a homeostatic mechanism in feed-forward (and also recurrent) networks that takes the inhomogeneities of the excitatory synaptic weight structure into account.

In a parallel study, Luz and Shamir (2012) have shown that the phenomenon is robust to the shape of the learning rule (Figures 1H,J,K). Asymmetric learning windows as observed by Haas et al. (2006), or even the classical asymmetric excitatory STDP (eSTDP) window (Gerstner et al., 1996; Markram et al., 1997; Bi and Poo, 1998; Song et al., 2000; Luz and Shamir, 2012) also lead to a stable balance of excitation and inhibition. The phenomenon is thus robust, as long as the learning rule obeys two fundamental requirements: Postsynaptic activity must predominantly potentiate activated inhibitory synapses, whereas in the absence of postsynaptic firing inhibitory synapses must decay. It is conceivable that the results observed by Haas et al. (2006) could also be fitted with a rule that is similar to the simplified rule proposed by Vogels et al. (2011), though with a maximum shifted by ~10 ms. Interestingly, the insensitivity of inhibitory plasticity to the exact shape of the learning window contrasts to the high sensitivity of excitatory plasticity to the formulation of the STDP curves, in particular the importance of the LTD window size relative to the LTP time window (Feldman, 2000; Song et al., 2000). This may be a consequence of the fact that inhibitory plasticity is mostly a negative feedback process, while excitatory plasticity is a form of positive feedback.

### Plasticity and dynamics of chloride reversal

Fast inhibition in the central nervous system is mainly mediated by chloride currents. To maintain inhibitory function, it is critical for cells to sustain a strong transmembrane Cl^−^ concentration gradient. Collapse of the hyperpolarized chloride reversal potential (Buzsáki et al., 2007; Blaesse et al., 2009), [e.g., through down-regulation of a neuron-specific K^+^/Cl^−^ co-transporter (KCC2)] is linked to pathologies such as chronic pain, schizophrenia, and epilepsy (Coull et al., 2003; Buzsáki et al., 2007; De Koninck, 2007; Kaila and Miles, 2010; Wamsteeker and Bains, 2010; Huberfeld et al., 2011). The regulation of the chloride reversal through development and its perturbation in pathological conditions has been extensively studied (Coull et al., 2003, 2005; Cordero-Erausquin et al., 2005; Rivera et al., 2005), but the emerging picture highlights some oversimplifying assumptions regarding the causes and consequences of shifting chloride reversal potential E^−^_Cl_.

E^−^_Cl_ has traditionally been thought of as effectively constant, changing only in a matter of several hours or days. Instead, the intracellular chloride concentration Cl^−^ is a dynamic quantity which, under some conditions, can drastically change in a matter of seconds or less (Staley et al., 1995; Staley and Proctor, 1999) and have specific, *local* effects on synaptic efficacy (Woodin et al., 2003; Raimondo et al., 2012). Consequently, robustness and kinetic time constant of Cl^−^ concentrations are as important as the baseline value. Further, in addition to the impact of cation-chloride co-transporter, the dynamic properties of Cl^−^ are also determined by the level of GABA-mediated activity, cell geometry, spiking and the homeostasis of other ionic species (Brumback and Staley, 2008; Fröhlich et al., 2008).

For example, in a study by Woodin et al. (2003), iSTDP was induced in hippocampal neurons by repetitively pairing pre- and postsynaptic action potential firing at a frequency of 5 Hz (for 150–300 pairings) (Woodin et al., 2003; Fiumelli and Woodin, 2007; Balena and Woodin, 2008; Saraga et al., 2008; Lamsa et al., 2010; Woodin and Maffei, 2010). When the firing was coincident (within ±15 ms), there was a decrease in the strength of GABAergic inhibition due to a depolarization of the reversal potential for GABA (E_GABA_). E_GABA_ depolarization resulted from a postsynaptic Ca^2+^ influx through L- and T-type voltage-gated Ca^2+^ channels (VGCCs) (Balena et al., 2010) which led to the decreased activity of KCC2 (Woodin et al., 2003). Essentially intracellular Cl^−^ was rising, and as a result decreased the driving force for Cl^−^ through GABA_A_ receptors. When action potential firing was non-coincident (>±50 ms), or for sole presynaptic spikes GABAergic synaptic transmission was weakened through a decrease in GABA_A_ receptor conductance (Figure 1I). Thus, while the time interval between pre- and postsynaptic spiking is important, the order of the spiking is not; this results in a symmetrical spike-timing window, which is in contrast to the asymmetric window for glutamatergic synapses in the same brain region (Bi and Poo, 1998). It should be noted here, that Figure 2 of Woodin et al. (2003) shows the identical rule as shown in Figure 1I, but recorded at a membrane potential more negative than E_GABA_. Figure 2 of Woodin et al. (2003) highlights a difference between two mechanisms that can modify synaptic efficacy: A change of E_GABA_, evoked by near coincident pre- and postsynaptic spikes (Δ*t* = [−40,+40]) and a change of synaptic conductance *g* caused by non-coincident spike pairs or presynaptic spikes alone. Because it is the difference between rest and reversal potential that determines the amplitude of the evoked postsynaptic current [plotted in Woodin et al. (2003)], and because we plot the change of synaptic efficacy from a presumed resting potential somewhere between E_GABA_ and threshold in Figure 1I, these two figures look different on first glance but express the same results. Two peculiarities set these results apart. First, Woodin and colleagues did not observe synaptic strengthening (relative to a resting potential >E_GABA_) in their protocol. Because synaptic weakening alone would ultimately abolish inhibitory transmission, the full synaptic plasticity rule may be more complex than described so far (see more below). Additionally, the results stress the importance of chloride reversal dynamics.

Since simultaneous control of all the factors influencing the Cl^−^ dynamics is difficult to achieve experimentally modeling becomes a privileged tool to study the spatiotemporal fluctuations of Cl^−^ and their consequences. Intricate models based on electrodiffusion instead of cable theory have been developed to account for fluctuations in ionic concentrations (Qian and Sejnowski, 1990; Bazhenov et al., 2004; Doyon et al., 2011). They show that in *in vivo*-like conditions even small changes in E^−^_Cl_ can have important functional consequences (Prescott et al., 2006). During high levels of joint excitatory and inhibitory activity, fast changes of only 5 mV in E^−^_Cl_ can have important functional implications on the input–output properties of a neuron because GABA activity can rapidly switch from depolarizing to hyperpolarizing, or vice versa.

Importantly, when disinhibition occurs through a loss of Cl^−^ extrusion capacity, e.g., by down-regulating chloride pumps or through high GABA activity, inhibitory efficacy cannot be restored through increasing GABA activity itself since such an increase would exacerbate the collapse of the Cl^−^ gradient. This has important implications for pain therapy, predicting that therapies aiming to restore Cl^−^ extrusion capacity or to mitigate the depolarizing bicarbonate current should be more efficient than those increasing GABAergic activity (De Koninck, 2007). In fact, the loss of Cl^−^ stability can lead to catastrophic failure through a positive feedback loop between Cl^−^ accumulation, membrane depolarization and spiking that turns inhibition into excitation. As excitation further depolarizes the membrane and thus increases the Cl^−^ driving force, slow Cl^−^ accumulation and progressive weakening of inhibition take place. Eventually spiking initiates and the subsequent membrane depolarization increases the Cl^−^ driving force further. These results predict that small doses of GABA-potentiating drugs are beneficial in restoring inhibition and why the observed therapeutic effect reaches a plateau as dosage is increased. They also explain why such drugs can become detrimental altogether for very high concentrations (Doyon et al., 2011). Experimentally, the response of the symptoms of pathological pain to the dosage of midazolam has been demonstrated to follow this pattern (Asiedu et al., 2010). Moreover, perturbation of the Cl^−^ gradient can also have consequences on the homeostasis of other ions because for example Cl^−^ influx through GABA channels occurs jointly with bicarbonate efflux [causing acidification of the cell (Staley and Proctor, 1999; Farrant and Kaila, 2007)], and Cl^−^ extrusion occurs jointly with K^+^ efflux (Krishnan and Bazhenov, 2011).

In summary, the effects of perturbing transmembrane Cl^−^ dynamics go beyond straightforward disinhibition, impacting the dynamic response of a neuron and the homeostasis of other ionic species. Moreover, even small shifts in E^−^_Cl_ cannot be discarded as irrelevant since they can be symptomatic of an underlying loss of robustness in the Cl^−^ gradient which could have important consequences in conditions of high level synaptic activity.

### Disinhibition-mediated excitatory LTP

The functional significance of iSTDP as observed by Woodin et al. (2003) has recently been demonstrated experimentally (Ormond and Woodin, 2009, 2011). In the hippocampus, the firing of presynaptic CA3 pyramidal neurons produces monosynaptic excitation of both CA1 pyramidal neurons and GABAergic interneurons. Excitation of these interneurons results in so called feed-forward inhibition onto those same pyramidal neurons, which can be so fast that excitation has not even reached its peak when inhibition begins to affect the postsynaptic membrane potential (Pouille and Scanziani, 2001; Ormond and Woodin, 2009). As a result, the inhibition shunts the preceding excitation, and prevents spiking. When GABAergic STDP is induced at these feedforward inhibitory inputs onto pyramidal neurons it produces a reduced shunting of excitatory synapses, resulting in long-term increases in the amplitude of Schaffer collateral-mediated postsynaptic potentials. This form of plasticity is called disinhibition-meditated eLTP (Ormond and Woodin, 2009) and can be summarized as a long-term, synapse-specific (Ormond and Woodin, 2011) increase in the amplitude of Schaffer collateral-mediated postsynaptic potentials. Like classic eLTP, disinhibition-mediated eLTP requires NMDAR activation, suggesting that it also plays a role in hippocampal-dependent learning and memory. It also suggests a tight co-regulation of excitatory and inhibitory plasticity.

## Plasticity of electrical synapses between inhibitory interneurons

Throughout the nervous system, interneurons are frequently connected to each other through both inhibitory and gap junctional (electrical) synapses (Galarreta and Hestrin, 2001; Connors and Long, 2004). Synchrony within interneuronal networks is likely to influence the network-wide effects of their output, i.e., the many inhibitory synapses that the interneurons in these networks make on pyramidal cells. Interneuronal network synchrony has been explored for simpler networks of cells coupled by both inhibitory and electrical synapses (Chow and Kopell, 2000; Lewis and Rinzel, 2003; Kopell and Ermentrout, 2004; Pfeuty et al., 2005; Saraga et al., 2006). In many cases, inhibitory and electrical synapses play complementary roles in coordinating the activity of neurons and their inhibitory output, although electrical synapses have been shown to act as inhibitory de-synchronizers for some neurons (Vervaeke et al., 2010).

Recent findings by Haas et al. (2011) demonstrate that synchronous bursting activity, a natural form of activity for thalamic neurons that is a component of sleep spindles, in pairs of coupled interneurons depresses the electrical synapse between them. This finding implies that levels of synchrony in coupled interneuronal networks may in turn be activity-dependent. In the thalamus, for example, the reduction in electrical coupling resulting from sleep spindles would desynchronize the thalamic reticular network, and as a result the inhibition sent back to thalamus would be less temporally structured or coordinated. The effects of dynamic variations in electrical synaptic strength on the coordination of interneuronal networks have yet to be explored, and may prove to be key modulators of the impact of inhibitory plasticity across the brain.

## Behavioral correlates of inhibitory plasticity

The potential functional implications of experience-dependent inhibitory plasticity on behavior are not often easy to untangle. A recent study attempts to explore a functional consequence of inhibitory plasticity in a mouse model of communication vocalization processing (Galindo-Leon et al., 2009; Lin et al., 2013). In this context, “inhibitory plasticity” refers not necessarily to *synaptic* inhibition (i.e., the release of GABA), but *functional* inhibition in which action potential generation is suppressed in a stimulus- and state-dependent manner. Mouse pups emit ultrasonic (60–80 kHz) vocalizations that are recognized as behaviorally relevant by mothers, but not by pup-naïve virgin females (Ehret, 2005). In both animal groups, well-isolated single units recorded from auditory cortex in awake, head-restrained animals can be inhibited by, excited by, or non-responsive to a library of natural ultrasonic calls played back at superthreshold intensities. The *average* call-evoked excitation is not significantly different between virgins and mothers, most likely because of a wide diversity of responses produces too much variability to decipher systematic changes from one group to another. However, calls evoking inhibition show a more uniform response across different calls, making it possible to reveal significant differences in the strength of evoked inhibition between mothers and virgins. Calls elicit deeper and longer inhibition in mothers compared to virgins; and importantly, this effect is most prominent for units in core auditory cortical fields tuned to sound frequencies more lateral to those found in the ultrasonic vocalizations themselves (i.e., <50 kHz).

What are the benefits of strengthened lateral band inhibition? Galindo-Leon and colleagues explain in the framework of labeled line propagation, in which a spiking neuron conveys information to downstream targets not just by its temporal pattern of action potentials, but also by which features that neuron represents. This is thought to be true in the core auditory cortex. Cells are coarsely arranged by their preferred sound frequencies in a tonotopic spatial arrangement. Action potentials from a neuron located along this tonotopic axis thus convey that the acoustic stimulus contains some stimulus feature around the corresponding frequency. Hence, to *read out* the frequency content of a sound, a downstream area could assess the relative firing from each “labeled line.” In this picture, the difference (i.e., contrast) of the activity between neuronal populations becomes important for the recognition of a stimulus. However, since excitatory tuning curves at superthreshold sound levels can have large bandwidths, this population representation might be broader than expected for a narrowband signal like an ultrasonic whistle call, thereby interfering with its recognition, particularly if the call occurs in broadband background noise (Ehret, 2005). On the other hand, if neural activity from best frequency bands *lateral* to the call frequency were more strongly inhibited, the population contrast would be enhanced, improving the recognition of ultrasound calls.

This state-dependent change in neuronal responsiveness may be due to the forms of cellular inhibitory plasticity discussed throughout this review. However, cortical neurons have varied and distinctly mosaïic projections that complicate the simplistic picture of labeled line propagation. Though recent results seem to support the existence of a population contrast mechanism in auditory and multi-modal integration (Cohen et al., 2011), future work in awake animals is critical, and detailed modeling of the mechanisms at hand will further illuminate the functional implications.

## Discussion

Brain networks and circuits respond to environmental stimuli and are shaped by them. The influence of experience on the connectivity and function of sensory areas of the brain has been investigated extensively (Hubel and Wiesel, 1963, 1970; Mower et al., 1982; Merzenich et al., 1983; Fox, 1992; Hofer et al., 2009; Wittenberg, 2010). While activity-dependent excitatory plasticity is relatively well characterized as a mechanism to control the (re-)wiring of cortical circuits, inhibitory plasticity presents a much less consistent set of rules. Considering the morphological, electrical and functional diversity of interneurons in the brain, this diversity of plasticity mechanisms is not surprising and will require the use of new techniques such as genetic targeting, but also computational modeling, to be understood.

The results presented at our workshop suggest several different functional roles for inhibitory plasticity. One class of learning rules is homeostatic in nature and maintains a balance of excitation and inhibition (Haas et al., 2006; Froemke et al., 2007; House et al., 2011; Vogels et al., 2011; Luz and Shamir, 2012): neurons that receive strong excitation will also receive strong inhibition, presumably to equalize the impact of all inputs to a cell, or to reduce the differences in neuronal output between neurons receiving strong and weak excitatory drive. A different class of rules (Maffei et al., 2006; Li et al., 2008) fosters competition between neurons or synaptic inputs by increasing the inhibitory drive in response to weak (mostly subthreshold) excitation, while allowing a veto of iLTP for strong (suprathreshold) excitation. Rules of this latter type thus act as contrast enhancers, in line with the behavioral results of Galindo-Leon et al. (2009). The observed iSTDP rules of Woodin et al. (2003) and Holmgren and Zilberter (2001) (Figures 1G,I, respectively) also fit into this class, in that they reduce inhibitory drive in response to coincident pre- and postsynaptic activity. Holmgren and Zilberter (2001), however, also found a potentiation of inhibitory synapses that are activated a few hundred milliseconds after the end of a postsynaptic action potential train, introducing additional complexity that is yet to be understood. Notably, inhibitory plasticity can also change the temporal structure of neuronal responses. In particular, Gilson et al. (2012) showed that iSTDP can shape the synaptic configuration of neurons such that they become selective to specific input oscillation frequencies.

It is important to mention that plasticity of excitatory or inhibitory synapses have mostly been studied as independent phenomena. A different picture emerges in recent work that investigates the impact of inhibition on excitatory plasticity. It is becoming increasingly clear that transient neuromodulatory changes in the balance of excitation and inhibition probably form an important factor for the induction of excitatory plasticity (see, e.g., Froemke et al., 2007; Letzkus et al., 2011), potentially providing a gating mechanism that would allow to selectively learn only “behaviorally relevant” stimuli. The studies of Ormond and Woodin (2009), Wang et al. (2012), and Paille et al. (2013) show that there is a complex interaction between inhibitory and excitatory synaptic plasticity that goes beyond the idea of neuromodulation of plasticity.

As inhibitory malfunction is often implicated in neuropsychiatric diseases, a better understanding of the dynamic regulation of inhibition could also provide new insights into the pathophysiological underpinnings of diseases such as epilepsy and schizophrenia. The development of new treatments will require a careful investigation of the underlying molecular machinery, such as biophysical controllers of Cl^−^ reversal (Woodin et al., 2003; Doyon et al., 2011).

The presented results highlight a synergetic interaction between experiment and theory in the field of inhibitory plasticity. The combination of experimental characterization of the plasticity of a given synapse type and subsequent computational modeling has proven successful in evaluating the functional purposes of inhibitory plasticity and promises to be a powerful tool for the large number of future studies that will be necessary until we understand the riddles of inhibitory function and plasticity.

### Conflict of interest statement

The authors declare that the research was conducted in the absence of any commercial or financial relationships that could be construed as a potential conflict of interest.
